# LDH-A Inhibitor as a Remedy to Potentiate the Anticancer Effect of Docetaxel in Prostate Cancer

**DOI:** 10.7150/jca.86283

**Published:** 2024-01-01

**Authors:** Cagri Cakici, Benay Daylan, Ruveyde Safiye Unluer, Ebru Emekli-Alturfan, Sule Ayla, Hilal Eren Gozel, Pakize Yigit, Elif Yavuz Dokgoz, Turkan Yigitbasi

**Affiliations:** 1 Department of Biochemistry, Faculty of Medicine, Istanbul Medipol University, Istanbul, Turkey, 34815.; 2 Department of Histology and Embryology, Faculty of Medicine, Istanbul Medipol University, Istanbul, Turkey, 34815.; 3 Graduate School of Health Sciences, Istanbul Medipol University, Istanbul, Turkey, 34815.; 4 Department of Basic Medical Sciences, Faculty of Dentistry, Marmara University, Istanbul, Turkey, 34857.; 5 Department of Histology and Embryology, Faculty of Medicine, Istanbul Medeniyet University, Istanbul, Turkey, 34720.; 6 Department of Medical Biology and Genetics, Faculty of Medicine, Istanbul Okan University, Istanbul, Turkey, 34959.; 7 Department of Biostatistics and Medical Informatics, Faculty of Medicine, Istanbul Medipol University, Istanbul, Turkey, 34815.; 8 Department of Biochemistry, Faculty of Pharmacy, Istinye University, Istanbul, Turkey, 34010.

**Keywords:** Prostate cancer, Apoptosis, EMT, LDH-A inhibitor, Warburg effect

## Abstract

Increased LDH-A activity promotes tumor growth, migration, invasion, and metastasis. This study aimed to investigate the effects of the combination of LDH-A inhibitor and Docetaxel on apoptosis and epithelial-mesenchymal transition (EMT) in the murine prostate cancer (PCa) model.

The prostate cancer murine model was developed subcutaneously in 50 male B57CL/6 mice using the Tramp-C2 prostate cancer cell line. From the tumor tissue samples, apoptosis analysis was performed using TUNEL staining, and EMT was investigated using western blot and qPCR. Hematoxylin-eosin staining (HE) and Periodic acid-Schiff staining were used to histopathologically examine liver and kidney tissues.

Lactate levels revealed that the Warburg effect was reversed with the LDH-A inhibitor. Both serum and tumor tissue apoptosis increased, and tumor sizes reduced in PCa+LDH-A inhibitor + Docetaxel treatment groups (p<0.05). The combination of LDH-A inhibitor and Docetaxel inhibited EMT mechanism by causing a decrease in Snail, Slug, Twist, and HIF-1α expressions as well as a decrease in N-cadherin and an increase in E-cadherin levels.

Reprogramming glucose metabolism with an LDH-A inhibitor can increase the effectiveness of Docetaxel on apoptosis and metastasis mechanisms in PCa.

## Introduction

Prostate cancer (PCa) is the most common malignancy in men worldwide and the second most common cause of cancer-related death following lung cancer 1. The metabolism of cancer cells varies compared to normal cells. The Warburg effect, which is an important phenomenon in cancer, describes the metabolic change in which cancer cells metabolize glucose preferentially through anaerobic glycolysis and produce lactate as the end product despite the presence of oxygen 2. LDH-A is the main form of LDH found in cancer cells which is the main regulator of anaerobic glycolysis. It actively reduces pyruvate levels and increases lactate levels in tumor tissues 3. Due to the increased lactate levels, the pH shifts towards acidity in the tumor microenvironment, which alters the tumor physiology. Increased LDH-A activity increases tumor growth, migration, invasion, and metastasis 4. Because of these features of LDH-A, combined treatments with inhibitors of LDH-A have recently become a topic of current research and a promising target in developing new anti-cancer agents 5.

A taxane derivative, Docetaxel is an effective anti-microtubule chemotherapeutic drug for first-line PCa treatment. In recent years, new chemotherapy regimens with synergistic effects that allow lower doses of chemotherapy have gained importance. Therefore, combined and comparative *in-vitro* and *in-vivo* studies of Docetaxel are important to develop new therapeutic agents for PCa 6.

Epithelial-to-mesenchymal transition (EMT) plays an important role in various biological and pathological processes, including wound healing, tissue fibrosis, and cancer progression 7. EMT is the most crucial step of metastasis in cancer cells. EMT is determined by revealing the changes in epithelial and mesenchymal markers. A decrease in epithelial markers (E-cadherin) and an increase in mesenchymal markers (N-cadherin, vimentin) indicate that the cell is undergoing EMT. Inhibition of EMT or conversion from EMT to mesenchymal-epithelial transition (MET) in cancer cells may be an effective strategy to prevent metastasis 8. Transcription factors such as SNAIL, SLUG, TWIST, play an essential role in cancer cells. All of these transcription factors are repressors of E-cadherin, which is the main component of intercellular connections, and are necessary for maintaining the mesenchymal state in cancer cells 9.

EMT induced by HIF-1α is important in metastasis. In cancer cells, acidosis induced by increased LDH-A activity causes resistance to apoptosis via HIF-1α 10, 11. Accordingly, increased LDH activity may be suggested to induce a more aggressive cancer character while promoting the invasiveness of tumor cells.

This study aimed to investigate the effect of LDH-A inhibitor + Docetaxel combination therapy on EMT and apoptosis in an *in-vivo* murine PCa model with an approach targeting cancer metabolism.

## Materials and Methods

### Cell culture experiment

Mouse Tramp-C2 PCa cell lines (ATCC® CRL-2731™) were used in the study. The Tramp-C2 PCa cell lines were cultured in an incubator at 37°C with 5% CO_2_, using a high-glucose Dulbecco's modified Eagle's medium (DMEM) cell culture medium, to which 5% fetal bovine serum (FBS), 5% Nu-serum IV, 10nM dehydroisoandrosterone (DHEA), 0.005 mg/ml bovine insulin and 1% penicillin-streptomycin was added.

### *In-vivo* prostate cancer model

In our study, 50 healthy 10-12 weeks old C57BL/6 type male mice obtained from Medipol Medical Research Center (MEDITAM) in İstanbul Medipol University were used. Mice were randomly divided into five groups. In the power analysis performed before the study, the presence of 6 animals in each group was found to be sufficient as it would give statistical significance with 84% power, and accordingly, each group consisted of 10 mice. Group 1: Phosphate buffer saline (PBS) treated Control group; Group 2: Tramp-C2 cells inoculated PCa Group; Group 3: 5 mg/kg Docetaxel treated PCa-Docetaxel Group; Group 4: LDH-A Inhibitor (Sodium Oxamate 300 mg/kg) treated PCa Group; Group 5: LDH-A Inhibitor + Docetaxel treated PCa group. LDH-A inhibitor injection was administered daily, and PBS and Docetaxel injections were administered intraperitoneally 3 times a week (Monday, Wednesday, and Friday) 12-17.

The mice were kept in 12 hours of light and 12 hours of darkness at room temperature of 22 ± 1˚C. No restrictions were placed on their diet. For this study, all respective procedures were approved by the Istanbul Medipol University Ethics Committee of Experimental Animal Use and the Research Scientific Committee (IMU-HADYEK) (Protocol 38828770-604.01.01-E.1507).

**Figure 1:**
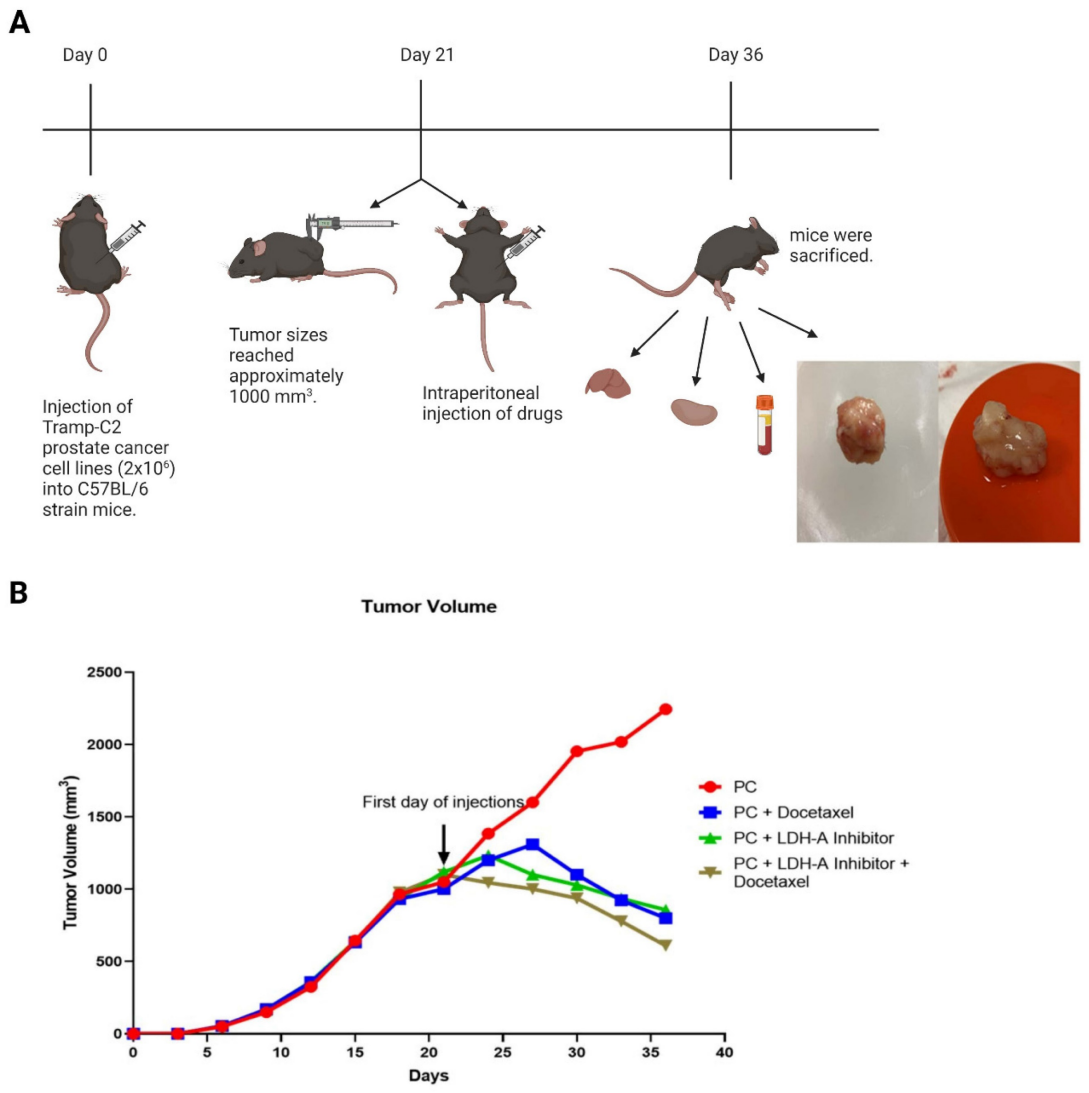
** (A)** Experiment flow chart. 50 healthy 10-12 weeks old C57BL/6 type male mice were used. Every group has 10 mice. 2x10^6^ cultured TRAMP-C2 prostate cancer cell line subcutaneously injected on the back of the mice. On the day 21, the tumor volumes reached approximately 1000 mm^3^ and the drugs were injected intraperitoneally (Group 1: Phosphate buffer saline (PBS); Group 2: PBS; Group 3: 5 mg/kg Docetaxel (6); Group 4: 300 mg/kg Sodium Oxamate (5); Group 5: 300 mg/kg Sodium Oxamate + 5 mg/kg Docetaxel). After treatment period, the mice were sacrificed, and tissues and blood samples were collected. The image was created with BioRender.com **(B)** Tumor volume change graph by days. Tumor volumes were measured every third day with using digital caliper and were calculated with using formula: Tumor Volume= (Length X Width^2^) /2. Created with BioRender.com.

Cultured Tramp-C2 cells were prepared in PBS as 2x10^6^ cells per mouse and inoculated subcutaneously on the back of the mice with a 27-gauge syringe. After the injection day, the tumor formation was monitored by palpating the area, and the tumor sizes reached approximately 1000 mm^3^ on day 21. Every day, the condition of the animals was monitored. The sizes of the tumors were measured with the help of a digital caliper every 3 days (Monday, Wednesday, and Friday), their volumes were calculated, and the experimental groups were formed (**Figure 1 A**). The formula for the calculation is as follows 12:

Tumor Volume= (Length X Width^2^) /2

### Collection of blood and tissue samples

Mice were anesthetized with ketamine/xylazine (200/10 mg/kg) after 15 days of drug administration. Blood was collected from the mice in red-capped gel blood tubes using the cardiac puncture method. The collected blood was centrifuged at 3000 rpm for 10 minutes, and serum samples were separated. Serum samples were stored at -80°C until assayed. Liver, kidney, and tumor tissues were also collected (**Figure 1 A**). While some of the tissues were stored at -80°C for biochemical analysis, the remaining tissues were placed in the neutral buffer solution for histological examinations and embedded in a paraffin block after tissue tracking.

### CK18-M30 and Lactate ELISA assays

Cytokeratin 18 (CK18), a type I intermediate filament protein and a part of the intracellular cytoskeleton, is released during the necrosis of both cancerous and healthy epithelial cells. If epithelial cells encounter apoptosis, caspase-mediated cleavage of CK18 leads to the breakdown of the intracellular cytoskeleton 18**.** Circulating full-length and caspase-cleaved CK18 is also considered as a biomarker of chemotherapy-induced cell death. Caspase-cleaved CK18 generated during apoptosis is measured by M30. Their potential as prognostic, predictive, and pharmacological strategies in cancer treatment has been recognized in prior research 19. Accordingly, serum apoptosis was determined using a Mouse CK18-M30 ELISA assay kit (Bioassay Technology Laboratory, Catalog No: E1994Mo). Serum Lactate concentrations were determined using the Mouse Lactate ELISA assay kit (MyBioSource, Catalog No: MBS756195).

### Histopathological examination

Tumor, liver, and kidney tissues removed from mice were placed in 10% neutral formalin, and then tissues were tracked. The tissues were embedded in paraffin block. TUNEL method was used to examine apoptosis in tumor tissue and hematoxylin-eosin (HE) staining and periodic acid-Schiff (PAS) staining were used to examine the damage in liver and kidney tissues, respectively.

### TUNEL method

Apoptosis was investigated in tumor tissues using the Roche Tunel kit (Roche 11684795910). DAPI staining was used to stain cell nuclei, and the prepared preparations were examined and visualized under a confocal immunofluorescence microscope.

### Hematoxylin-eosin staining (HE)

HE staining was performed to evaluate the general toxicological effects of our treatments on histological changes in mouse liver tissues. At the end of the study, liver tissues from mice were fixed in 10% formalin solution (Tekkim). Tissues were maintained at 60°C in an ascending ethanol (Sigma-Aldrich Corp.) series (70-100%) for dehydration. Then, the transparency process was applied with two xylene (Tekkim) exchanges. The tissues were kept at room temperature for 1 hour. In the final step, all tissue samples were embedded in paraffin (Tekkim) blocks, cut into 5 μm slices, and stained with HE (Bio-Optica) for histological examinations.

### Periodic acid-Schiff Staining (PAS)

At the end of the study, kidney tissues taken from mice were fixed in the 10% formalin solution (Tekkim) and embedded in paraffin. Sections (5 μm) were stained with PAS and examined double-blindly. At least 10 areas were examined for each kidney section and scored for pathological injury. A score of 0 to 4 was assigned for pathological assessment: 0, normal histology; 1, mild injury, 5% to 25% of tubules showing pathological damage; 2, moderate injury, 25% to 50% of tubules showing pathological damage; 3, serious injury, 50% to 75% pathological damage; and 4, damage to nearly all tubules in its entire area. The mean histological score was calculated for each sample 20. Images were taken on a Nikon Eclipse Ni microscope (Nikon Instruments Inc., Melville, NY, USA).

### Evaluation of EMT mechanism using Western blot method

The tumor tissue samples (5 mg) were lysed entirely in cold RIPA lysis buffer, and tissue lysates were kept on the orbital mixer at +4°C for 2 hours. The protein concentration of the supernatant was determined, and samples containing 20-30 μg of protein were run in BIORAD brand gel with 4-20% percentage with loading buffer under 100-150 V for 1 hour in sodium dodecyl sulfate polyacrylamide gel electrophoresis (SDS-PAGE). Proteins on the gel were transferred onto a polyvinylidene fluoride (PVDF) membrane. Membrane was blocked with EveryBlot Blocking Buffer (BioRad) was incubated with primary antibodies E-cadherin (1:1000; BT-lab, China), N-cadherin (1:1000; Elabscience, US), Snail (1:1000; BT-lab, China), Slug (1:1000; Elabscience, US), Twist (1:1000; BT-lab, China), HIF-1α (1:1000; BT-lab, China), and β-Actin (1:3000; BT-lab, China) overnight at +4˚C. Membranes treated with primary antibodies were washed 3 times with tris buffer-Tween 20 (TBST) solution and incubated for 1 hour at room temperature with HRP-conjugated secondary antibodies. Proteins were detected using a Bio-Rad ChemiDoc XRS + System, and the bands were quantified using the Image J program.

### Evaluation of EMT mechanism using RT-qPCR

Total RNA was isolated using RNA isolation kit (GeneALL Seoul, Korea) and complementary DNA (cDNA) synthesis was performed using the ABT cDNA synthesis kit (ABT, Turkey) following the manufacturer's protocol. RT-qPCR was then performed using a PCR instrument (ABI, Foster City, CA, USA), and in each RT-qPCR, three biological replicates were used, each in triplicate (n = 3). E-cadherin (Mm01247357_m1, Taqman Probes, Thermo), N-cadherin (Mm01162497_m1, Taqman Probes, Thermo), Snail (Mm00441533_g1, Taqman Probes, Thermo), Slug (Mm00441531_m1, Taqman Probes, Thermo), Twist (Mm00442036_m1, Taqman Probes, Thermo) and HIF-1α (Mm00468869_m1, Taqman Probes, Thermo) expression levels were normalized to Eukaryotic 18S rRNA Endogenous Control (4333760T, Thermo). Fold changes in expression were calculated by using 2^-ΔΔCt^. ΔCt represents the difference between each target's threshold cycle (Ct) and housekeeping mRNA.

### Statistics

Statistical analysis was performed using GraphPad Prism 8.0 (GraphPad Software, Inc., La Jolla, CA, USA) and SPSS version 22.0 (SPSS, Chicago, IL, United States). One-way analysis of variance (ANOVA) was used to evaluate the differences between the groups within the scope of the evaluated parameters, and Tukey's multiple comparison test was used as the post-hoc test. The *p* ≤0.05 level was considered statistically significant.

## Results

The study, which started with 50 animals, was terminated with 33 animals when 17 animals died. There was no loss of animals in the Control Group. In the PCa control group, 5 animals, PCa-Docetaxel group 4 animals, PCa-LDH inhibitor group 4 animals, PCa-LDH inhibitor + Docetaxel group 4 animals died during the experiment.

### Tumor volume change

Tumor sizes reached approximately 1000 mm^3^ at day 21 in the Tramp-C2 PCa cell lines injected C57BL/6 type mice. The drugs were started to be administered from the 21st day on the mice. When the tumor volumes at the beginning and end of the treatment were compared, they were found to increase (1050 -2245 )1195 mm^3^ in the PCa Control Group, while the tumor volumes in the treatment groups decreased. Decreases from the beginning of treatment were 202 mm^3^ (1002-800 mm^3^) in the PCa- Docetaxel group, 262 mm^3^ (1119-857 mm^3^) in the PCa- LDH-A inhibitor group, and 379 mm^3^ (1100-721 mm^3^) in the PCa-LDH-A inhibitor +Docetaxel group. At the end of day 36, while the tumor volume continued to increase in the PCa control group, it was observed that the tumor volume of the Docetaxel and LDH-A inhibitor groups decreased compared to the control group. The lowest tumor volume was detected in the LDH-A inhibitor + Docetaxel combination group (**Figure 1 B**).

### Results of apoptosis analysis using CK18-M30 ELISA test and TUNEL method

**Figure 2:**
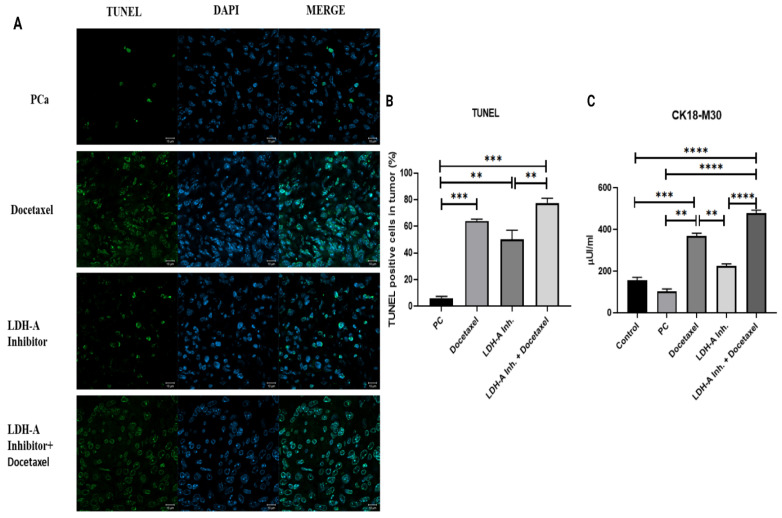
** (A)** Microscopy images of cells that underwent apoptosis by the TUNEL method in tumor tissues from mice. **(B)** Bar graph with % of cells apoptosis by the TUNEL method. **(C)** Bar graph of CK18-M30 apoptosis marker analyzed by ELISA method in serum. * p < 0.05, ** p < 0.01, *** p < 0.001, **** p < 0.0001 were considered significant.

The apoptosis results analyzed by the TUNEL method in tumor tissues revealed that the percentage of cells undergoing apoptosis in the PCa group was the lowest. Noticeable apoptotic cell densities were observed in the PCa+Docetaxel group and PCa+LDH-A inhibitor group, while the highest number of apoptotic cells was observed in the PCa+LDH-A inhibitor + Docetaxel combination group. When compared with the control group, the percentages of cells undergoing apoptosis increased significantly in the PCa+Docetaxel, PCa+LDH-A inhibitor, and PCa+LDH-A inhibitor+Docetaxel combination groups (p < 0.001, p < 0.01, and p < 0.001 respectively) **(Figure 2 A-B)**.

CK18-M30 levels in serum evaluated using the ELISA method showed parallelism with the results of the TUNEL analysis. Although statistically not significant, CK18-M30 levels in the PCa group were lower when compared with the control group. Serum CK18-M30 levels increased significantly in the PCa+LDH-A inhibitor+Docetaxel group when compared to the PCa (p < 0.0001), PCa+LDH-A inhibitor (p < 0.0001) **(Figure 2 C)**.

### Results of serum lactate analysis

Lactate levels were measured to examine the effectiveness of the LDH-A inhibition (Sodium Oxamate) and to understand the effects of the treatments on the Warburg effect.

**Figure 3:**
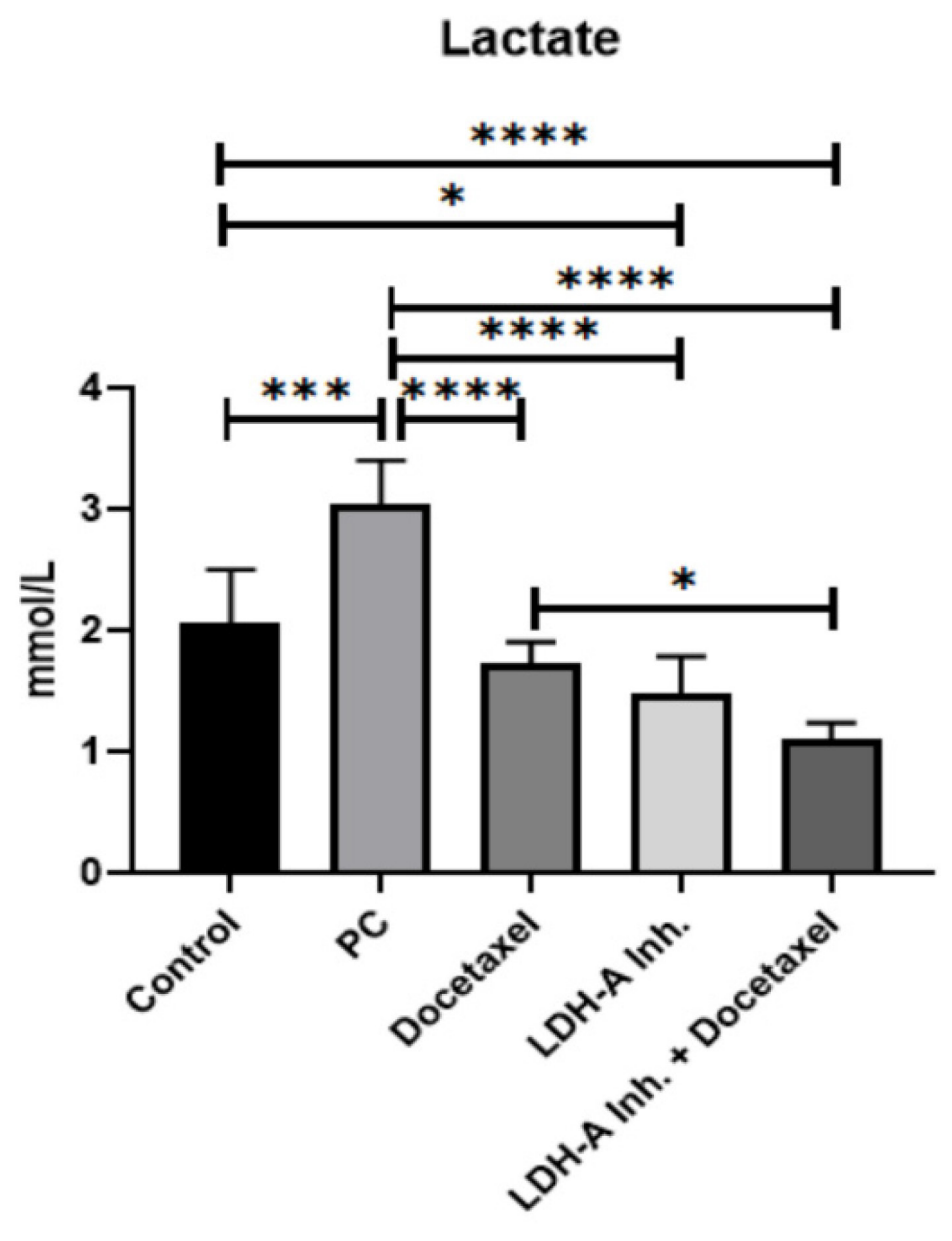
Results of serum lactate analysis. * p<0.05, ** p<0.01, *** p<0.001, **** p<0.0001 were considered significant.

As cancer cells use more anaerobic glycolysis than normal cells, LDH-A enzyme activities are very high, and the amount of lactate formed in the cell increases. Accordingly, our study observed the highest lactate concentration in the PCa group, as expected. Docetaxel, LDH-A inhibition, and LDH-A inhibition+Docetaxel treatments decreased lactate levels in the PCa groups (p < 0.0001). Although LDH-A inhibitor reduced lactate levels, sodium oxamate did not affect other isoforms of LDH, and complete inhibition was not achieved. The lowest lactate levels were observed in the LDH-A inhibitor + Docetaxel combination group **(Figure 3)**.

### Investigation of toxicity in kidney and liver tissues

**Figure 4:**
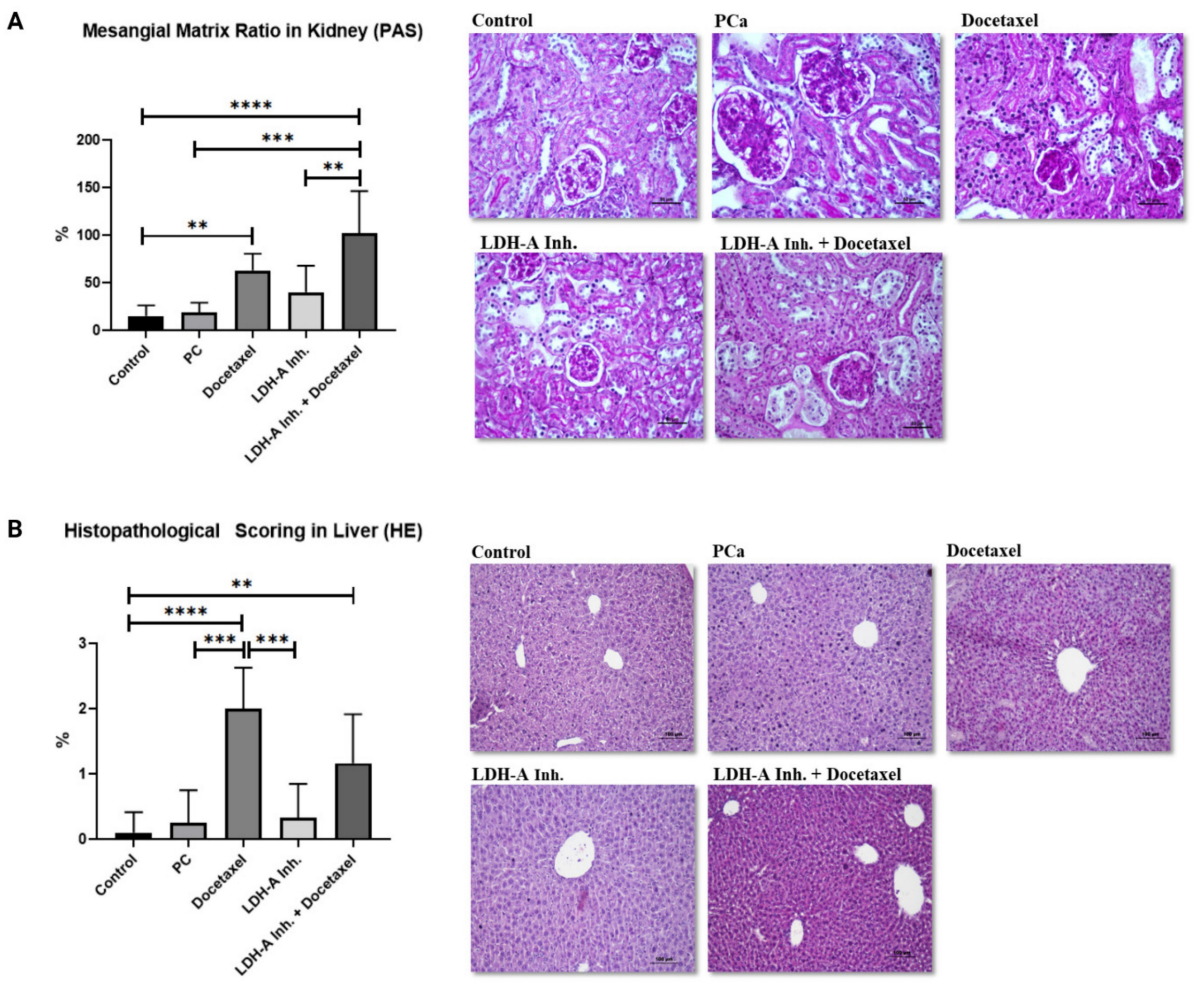
** (A)** Evaluation of mesenchial matrix ratio in kidney tissues with PAS staining. **(B)** Histopathological scoring in liver tissues by HE staining. * p<0.05, ** p<0.01, *** p<0.001, **** p<0.0001 were considered significant.

The ratio of glomerular mesangial matrix in the kidney revealed by PAS staining was found to be statistically higher in PCa+Docetaxel and PCa+LDH-A inhibitor + Docetaxel combination groups compared to the control group (p < 0.0001 and p < 0.01 respectively). In addition, kidney damage in the PCa+LDH-A inhibitor+Docetaxel group was statistically higher than the PCa group (p < 0.0001) and PCa+LDH-A inhibitor group (p < 0.01) **(Figure 4A)**. Despite numerical differences, no statistical significance was found between the other groups. There were no abnormal findings in the kidney sections of the control and PCa groups and the group that received LDH-A inhibitor treatment. Glomerular basement membrane and mesangial matrix ratios were normal in this group **(Figure 4 A)**. An increase in the mesangial matrix was observed in the kidney sections of the group treated with Docetaxel and LDH-A inhibitor+Docetaxel, hypertrophy and hyperplasia were observed in the bowman capsule parietal leaf cells, and hypertrophy was observed in the glomeruli **(Figure 4 A)**.

In the histopathological scoring of the liver with HE staining, significantly increased damage was found in the Docetaxel and LDH-A inhibitor + Docetaxel treatment groups compared to the control group **(**p < 0.0001 and p < 0.01 respectively**)**. Liver damage in the PCa group was found to be significantly lower than the Docetaxel treatment group (p < 0.001). Liver damage in the docetaxel treatment group was statistically higher than in the LDH-A inhibitor treatment group (p < 0.001). Although significantly increased liver damage was observed in the Docetaxel group compared to the PCa group, combination treatment with LDH-A inhibitor+ Docetaxel led to reduced liver damage. There were no abnormal findings in the liver sections of the control, PCa group, and LDH-A inhibitor treatment groups. In this group, a natural-looking liver parenchyma was observed, hepatocytes were observed as radial cell cords around the central vein, and sinusoids were normal **(Figure 4 B)**. A mild degeneration was observed in the liver parenchyma of the LDH-A inhibitor+Docetaxel treated PCa group. Hepatocyte cords were irregular, there was a slight increase in mononuclear cell infiltration, dilatation was observed in the sinusoids, and the central vein lost its structure **(Figure 4 B)**.

### Results of EMT analysis in tumor tissues

A decrease in the epithelial marker E-cadherin and an increase in the mesenchymal marker N-cadherin indicate that the cell has undergone EMT. EMT is also associated with an increase in the EMT transcription factors Snail, Slug, and Twist, which are suppressors of E-cadherin. Examination of EMT parameters is critical to understand whether the cell has metastasized. We analyzed HIF-1α, the protein of the hypoxia pathway and one of the EMT signaling pathway components, to examine tumor cells' resistance against the treatments used.

**Figure 5:**
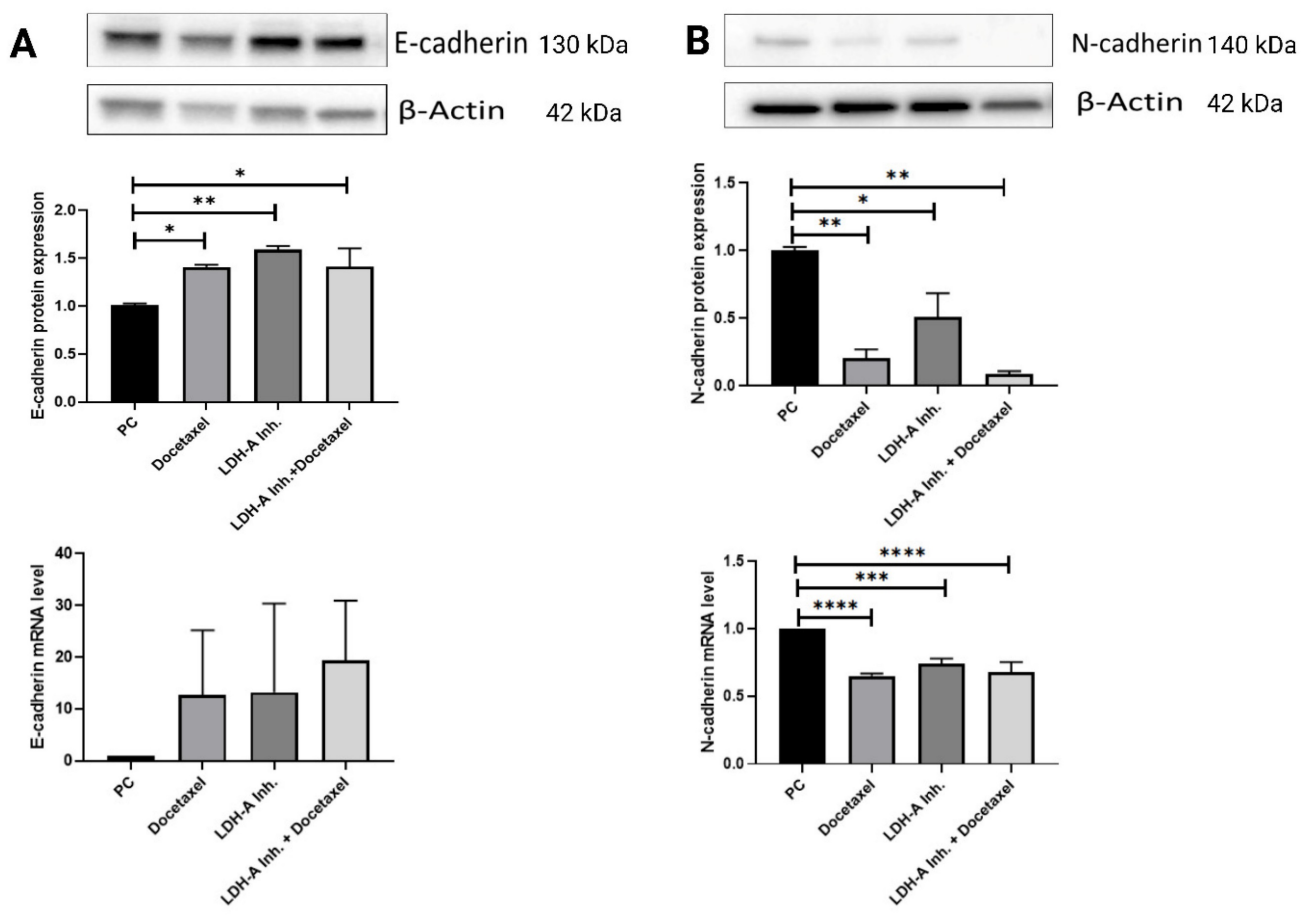
** (A)** Western blot and qPCR results of E-cadherin protein. E-cadherin is an epithelial marker for EMT. Decreases in E-cadherin responsible for loosing cell's epithelial characteristic. In this study it was important to show how the combined therapy effects on EMT markers **(B)** Western blot and qPCR results of N-cadherin protein. N-cadherin is a mesenchymal marker. Increases in N-cadherin shows that cancer cell gains metastatic characteristic. * p<0.05, ** p<0.01, *** p<0.001, **** p<0.0001 were considered significant. Created with BioRender.com.

E-cadherin protein expressions in the PCa+Docetaxel, PCa+LDH-A inhibitor, and PCa+LDH-A inhibitor + Docetaxel treatment groups increased significantly compared to the PCa group (p < 0.05, p < 0.01 and p < 0.05 respectively**)**. E-cadherin mRNA expressions of the PCa group was found to be lower than that of the treatment groups, confirming the western blot results **(Figure 5 A)**.

N-cadherin protein expressions in tumor tissue decreased significantly in the PCa+Docetaxel, PCa+LDH-A inhibitor, and PCa+LDH-A inhibitor + Docetaxel groups compared to the PCa group (p < 0.01, p < 0.05 and p < 0.01 respectively). mRNA levels of PCa+Docetaxel, PCa+LDH-A inhibitor, and PCa+LDH-A inhibitor + Docetaxel groups were found to be statistically lower compared to the PCa group (p < 0.0001, p < 0.001 and p < 0.0001 respectively)** (Figure 5 B).**

**Figure 6:**
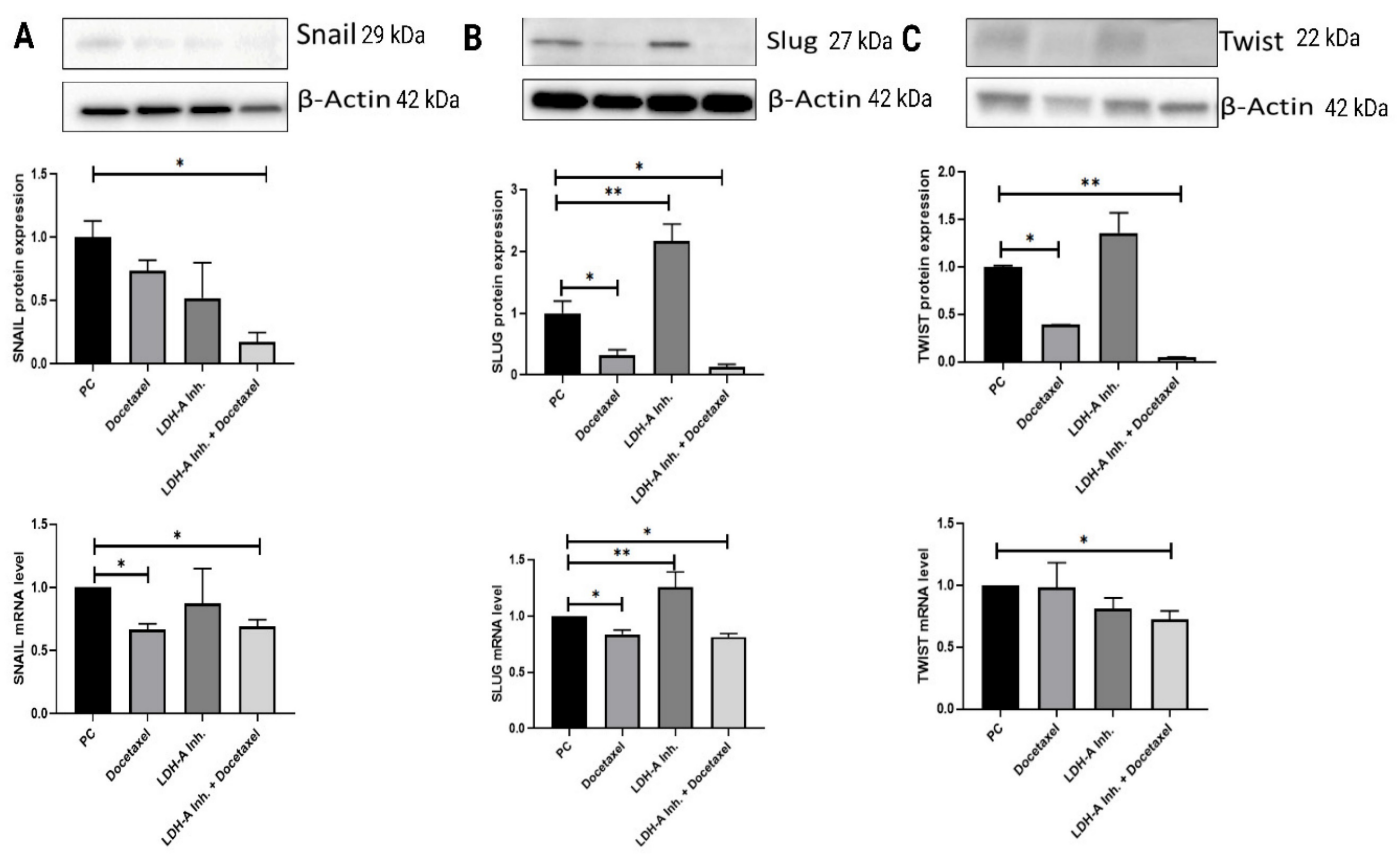
** (A)** Western blot and qPCR results of Snail protein. **(B)** Western blot and qPCR results of Slug protein. **(C)** Western blot and qPCR results of Twist protein. The transcription factors are responsible for suppression of E-cadherin. In this experiment 5 groups were analyzed and the differences between groups were analyzed with using One-Way Anova test. *p<0.05, ** p<0.01, *** p<0.001, **** p<0.0001 were considered significant. Created with BioRender.com.

When Snail expression in tumor tissue was examined, Snail expressions were found to be significantly lower in the PCa+LDH-A inhibitor + Docetaxel treatment group compared to the PCa group (p < 0.05). The decreases in PCa+Docetaxel and PCa+LDH-A inhibitor treatment groups were not statistically significant. Snail mRNA levels of the PCa+Docetaxel and PCa+LDH-A inhibitor + Docetaxel treatment groups were found to be significantly lower than the PCa group (p < 0.05). The decrease in the Snail mRNA levels of the PCa+LDH-A inhibitor group was not statistically significant **(Figure 6 A)**.

When Slug protein western blot expressions were analyzed, the PCa+LDH-A inhibitor treatment group's Slug expression was significantly higher than that of the PCa group (p < 0.05). Slug expression of the PCa+Docetaxel and the PCa+LDH-A inhibitor+Docetaxel groups decreased significantly compared to the PCa group (p < 0.05). When the slug mRNA levels were examined, the Slug mRNA levels of the PCa+Docetaxel and PCa+LDH-A inhibitor+Docetaxel treatment groups were found to be significantly lower than the PCa group (p < 0.05). The mRNA levels of the PCa+LDH-A inhibitor treatment group increased significantly compared to the PCa group (p > 0.01) **(Figure 6 B)**.

Twist protein expressions in the tumor tissues increased significantly in the PCa+Docetaxel and PCa+LDH-A inhibitor + Docetaxel treatment groups when compared to the PCa group **(**p < 0.05 and p < 0.01, respectively**)**. Twist mRNA levels of the PCa+LDH-A inhibitor + Docetaxel group were found to be significantly lower than the PCa group (p < 0.05). The decrease in the Twist mRNA levels of the PCa+LDH-A inhibitor treatment group was not statistically significant **(Figure 6 C)**.

**Figure 7:**
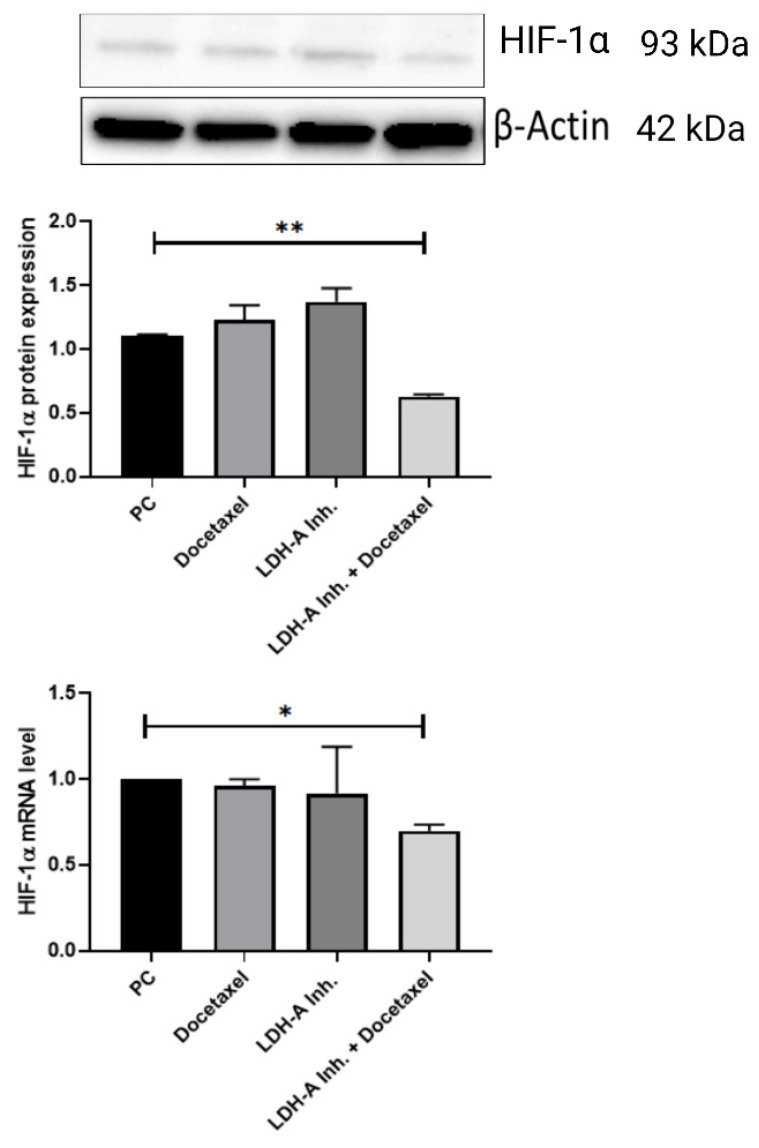
Western blot and qPCR results of HIF-1α protein. HIF-1α concentration is important to understand docetaxel resistance in prostate cancer. Because increasing HIF-1α protein concentration and mRNA level shows shows parallelism with docetaxel resistance. *p<0.05, ** p<0.01, *** p<0.001, **** p<0.0001 were considered significant. Created with BioRender.com.

It is known that long-term use of Docetaxel in PCa causes chemotherapeutic drug resistance. When tumor cells become resistant to Docetaxel, HIF-1α protein expression increases, and this is thought to be a mechanism of resistance to therapy for the cell. We examined HIF-1α protein and mRNA expression levels to examine the chemoresistance status of the treatments we applied in PCa. HIF-1α expressions in the tumor tissues decreased significantly in the PCa+LDH-A inhibitor + Docetaxel treatment group compared to the PCa group (p < 0.01). When the HIF-1α mRNA levels were analyzed, they were found to be significantly lower in the PCa+LDH-A inhibitor + Docetaxel treatment group compared to the PCa group (p < 0.05) **(Figure 7)**.

## Discussion

PCa is the second most common type of cancer in men worldwide, and cases of PCa are increasing in developed countries 21. Treatment of PCa continues to be a significant issue due to increasing mortality rates. There are different treatment options for PCa. One of them, Docetaxel, is a taxane derivative that acts by binding to microtubules and preventing androgen receptor nuclear translocation and causing apoptosis through B cell lymphoma (Bcl-2) phosphorylation 22. However, clinical and experimental studies have revealed that long-term administration of Docetaxel in PCa causes chemotherapy resistance 23, 24.

LDH-A catalyzes the conversion of pyruvate to lactate in anaerobic glycolysis, and its activity increases excessively in human cancer types such as hepatocellular, breast, and prostate. LDH-A inhibition has been suggested to delay tumor formation and progression 4. In recent years, new chemotherapy regimens with synergistic effects that enable lower doses of chemotherapy have gained importance 6. Accordingly, we investigated the effects of the combination of Docetaxel, which is used as a conventional treatment, with an LDH-A inhibitor in the *in-vivo* PCa model.

Apoptosis, defined as programmed cell death, is observed in normal physiological processes such as embryogenesis and adult tissue homeostasis. Apoptosis also acts as a tumor suppressor mechanism in cancer cells 25. Studies have shown that Docetaxel and LDH-A inhibitors induce apoptosis both *in-vivo* and *in-vitro* 26-31. Our study showed that Docetaxel and LDH-A inhibitor-induced apoptosis in serum and tumor tissue.

In addition, we demonstrated that adding an LDH-A inhibitor to the treatment potentiates the effect of Docetaxel on apoptosis and reduces tumor sizes in the *in-vivo* model of PCa. We interpreted this effect as the result of the regulation of energy metabolism by LDH-A inhibitors.

LDH-A is abnormally elevated in many types of cancer and promotes metabolic reprogramming and growth, malignant proliferation, and metastasis of cancer cells. Therefore, it acts as the checkpoint of anaerobic glycolysis in cancer cells 32. In a study by Yamada et al., it was reported that high serum LDH levels in PCa adversely affect the prognosis of the disease 33. In the study of Nunes et al., it was observed that glycolysis and lactate production increased in advanced PCa, and therefore PCa progressed more aggressively 34. Similarly, in our study, LDH-A specifically showed a positive effect on the progression of prostate cancer with sodium oxamate. It was observed that lactate levels decreased in the LDH-A inhibitor group, but there was still lactate production. This is because the sodium oxamate is specific inhibitor to LDH-A, and it has been thought that other LDH isoenzymes maintain a certain amount of lactate production. So, inhibiting LDH-A, specifically with sodium oxamate, had a positive impact on the advancement of prostate cancer.

The study of Hiew et al. observed that Docetaxel treatment in PCa cell lines decreased LDH-A and lactate levels 35. Our study observed that separately Docetaxel and LDH-A inhibitor decreased lactate levels, but the lowest lactate level was in the PCa+LDH-A inhibitor + Docetaxel combination group. Depending on this effect, combination therapy can be considered a treatment option in regulating the cell's energy metabolism by reversing the Warburg effect.

Docetaxel has long been used to treat various types of cancer, including glioblastoma, breast, and prostate. However, it causes many side effects in normal tissues such as the brain and testis. In our study, kidney damage in the PCa+LDH-A inhibitor+Docetaxel group was statistically higher than the PCa group and PCa+LDH-A inhibitor group. Docetaxel is excreted via the kidneys. Therefore, Docetaxel causes nephrotoxicity as a side effect in human kidney cells. The reason for the nephrotoxic effect of Docetaxel is still a subject of research. Bas et al.'s study showed that the toxicity of Docetaxel was induced by excessive mitochondrial ROS production 36. In a study by Yarim et al., it was shown that a dose of 30 mg/kg of Docetaxel increased oxidative stress in the body and created toxicity related to oxidative stress in brain and liver tissues 37. Since Docetaxel is a nephrotoxic chemotherapeutic agent, damage to the mesangial matrix was increased histologically in the kidney only in the PCa+Docetaxel group, while this histological damage was more in the PCa+LDH-A inhibitor + Docetaxel combined treatment group. It is known that inhibition of LDH-A induces oxidative stress 38. This suggests that the oxidative stress increasing effect of Docetaxel is enhanced by the LDH-A inhibitor. However, in the liver, the use of LDH-A inhibitor together with Docetaxel had a reducing effect on liver damage. Determining the drug dose that causes the least kidney damage by using different dose ranges of Docetaxel + LDH-A inhibitor may be the target of our next study.

EMT is a process in which epithelial cells lose cell polarity, cell-cell adhesion, and gain migration and invasion properties. EMT-related transcription factors such as Twist, Snail, and Slug are all required for migration. E-cadherin, as the key epithelial marker responsible for adhesion junction, enables cells to maintain their epithelial phenotype. In our study decreased expression of E-cadherin and increased N-cadherin are associated with metastatic progression of PCa. Therefore, these EMT markers have the potential to be important prognostic factors in predicting the course of PCa 39. Our study found that the protein expression of E-cadherin was significantly higher in all treatment groups compared to the prostate cancer group, and at the same time, mRNA levels were also found to be numerically high. On the other hand, N-cadherin's protein expression and mRNA levels were found to be statistically significantly low in all treatment groups compared to the prostate cancer group. It was shown that EMT was inhibited in all treatment groups, especially in the combined group.

EMT transcription factors (Snail, Slug, Twist) initiate metastatic mechanisms by suppressing E-cadherin in malignant tumors 40. Studies have shown that prolonged use of Docetaxel treatment alone in prostate cancer, breast cancer, lung cancer, and ovarian cancer undesirably increases the protein concentrations of Snail, Slug, and Twist 41-44. A study conducted by Hou and colleagues on lung cancer showed that the decrease in LDH-A expression inhibits the EMT mechanism 44. In our study, for the first time, the effects of the LDH-A inhibitor+docetaxel combination were investigated in prostate cancer murine model, and it was found that adding the LDH-A inhibitor to Docetaxel had a positive effect on both protein expression and mRNA levels, statistically significantly decreasing the Snail, Slug, and Twist transcription factors.

In recent years, Docetaxel has been accepted as the standard first-line therapy in PCa cases. However, Docetaxel resistance developed as a result of treatment provides a limited survival advantage 45. Studies have shown that Docetaxel, when used for a long time in cancer types such as PCa, breast cancer, lung and ovarian cancer, causes drug resistance after a certain period of time and this develops due to EMT 46-49. LDH-A is one of the key enzymes for glycolysis, which is one of the energy pathways. Overexpression of LDH-A is observed in solid tumors, which is thought to be associated with tumor progression 50-52. LDH-A has been shown to increase with the increase of HIF-1α concentration in PCa 41, 53. In addition, it was observed that a correlated increase in LDH-A and HIF-1α caused drug resistance in myeloma and PC3-RR (radio resistance prostate cancer cell line) cancer cells 29, 42. This suggests that the effect of LDH-A inhibition on EMT is through the hypoxia pathway. Previous studies have shown that the complete inhibition or the decrease in the concentration of LDH-A is sufficient for the inhibition of cell growth and EMT, which plays a significant role in metastasis 43, 44.

In our study, an increase in E-cadherin levels and a decrease in N-cadherin levels were observed in the treatment groups. In particular, the combined treatment group was observed to be the most effective group. In addition, when HIF-1α results were examined, a slight increase was observed in the PCa+Docetaxel and PCa+LDH-A inhibitor groups at the end of the 15-day treatment period, while a decrease was observed in the combined treatment group. This makes us think that the combined use of LDH-A inhibitor and Docetaxel is effective in converting the EMT mechanism into MET and can prevent drug resistance due to Docetaxel since Docetaxel administered alone causes drug resistance after a certain period. In order to prevent metastasis, hypoxic environment and angiogenesis must be prevented. Based on our results, the combined therapy applied in our study may be suggested to be effective in converting the hypoxic environment to the normoxic environment and may be suggested for EMT to MET transition. Moreover, the decrease in the E-cadherin suppressor transcription factors Snail, Slug, Twist reveals that the most effective treatment for the inhibition of the metastasis mechanism is the combined treatment.

## Conclusion

As a result, our results showed that the treatment groups induced apoptosis and reduced tumor volume. It was observed that the combined treatment increased E-cadherin by suppressing EMT transcription factors (Snail, Slug, Twist), thus being most effective in the conversion of EMT to MET. LDH-A inhibition and decreased HIF-α in the combined therapy group are likely to prevent EMT by inhibiting Docetaxel drug resistance. Therefore, our results showed that LDH-A inhibition enhanced Docetaxel's efficacy in therapy and suggested that LDH-A inhibition could be a new approach to treating PCa.
